# Analyzing Relationships Between Economic and Neighborhood-Related Social Determinants of Health and Intensive Care Unit Length of Stay for Critically Ill Children With Medical Complexity Presenting With Severe Sepsis

**DOI:** 10.3389/fpubh.2022.789999

**Published:** 2022-04-29

**Authors:** Hunter Hamilton, Alina N. West, Nariman Ammar, Lokesh Chinthala, Fatma Gunturkun, Tamekia Jones, Arash Shaban-Nejad, Samir H. Shah

**Affiliations:** ^1^Department of Pediatrics, Division of Pediatric Critical Care Medicine, University of Tennessee Health Science Center College of Medicine, Memphis, TN, United States; ^2^University of Tennessee Health Science Center – Oak-Ridge National Laboratory Center for Biomedical Informatics, Department of Pediatrics, College of Medicine, Memphis, TN, United States; ^3^Clinical Trials Network of Tennessee, University of Tennessee Health Science Center, Memphis, TN, United States; ^4^Departments of Pediatrics and Preventive Medicine, University of Tennessee Health Science Center College of Medicine, Memphis, TN, United States; ^5^Children's Foundation Research Institute Biostatistics Core, Memphis, TN, United States

**Keywords:** health disparities, social determinants of health, children with medical complexity, severe sepsis, pediatric intensive care unit

## Abstract

**Objectives:**

Of the Social Determinants of Health (SDoH), we evaluated socioeconomic and neighborhood-related factors which may affect children with medical complexity (CMC) admitted to a Pediatric Intensive Care Unit (PICU) in Shelby County, Tennessee with severe sepsis and their association with PICU length of stay (LOS). We hypothesized that census tract-level socioeconomic and neighborhood factors were associated with prolonged PICU LOS in CMC admitted with severe sepsis in the underserved community.

**Methods:**

This single-center retrospective observational study included CMC living in Shelby County, Tennessee admitted to the ICU with severe sepsis over an 18-month period. Severe sepsis CMC patients were identified using an existing algorithm incorporated into the electronic medical record at a freestanding children's hospital. SDoH information was collected and analyzed using patient records and publicly available census-tract level data, with ICU length of stay as the primary outcome.

**Results:**

83 encounters representing 73 patients were included in the analysis. The median PICU LOS was 9.04 days (IQR 3.99–20.35). The population was 53% male with a median age of 4.1 years (IQR 1.96–12.02). There were 57 Black/African American patients (68.7%) and 85.5% had public insurance. Based on census tract-level data, about half (49.4%) of the CMC severe sepsis population lived in census tracts classified as suffering from high social vulnerability. There were no statistically significant relationships between any socioeconomic and neighborhood level factors and PICU LOS.

**Conclusion:**

Pediatric CMC severe sepsis patients admitted to the PICU do not have prolonged lengths of ICU stay related to socioeconomic and neighborhood-level SDoH at our center. A larger sample with the use of individual-level screening would need to be evaluated for associations between social determinants of health and PICU outcomes of these patients.

## Introduction

Children with medical complexity (CMC) are frequently described as having multiple chronic conditions, resulting in functional limitations, ongoing use of medical technology, and high resource utilization (1–3) and account for 0.4% of children in the United States (1). In addition to chronic comorbidities, CMC are disproportionately affected by acute critical illness, including severe sepsis (4). Severe sepsis can lead to increased pediatric hospitalizations (5–7), possibly further affecting this population. CMC have more admissions and greater morbidity and mortality when compared with previously healthy children (4, 8–10). Social determinants of health (SDoH) related to socioeconomic status (poverty level, household income) and neighborhood environment, including housing instability (especially household crowding), neighborhood quality, and access to food, transportation, and healthcare (11), may lead to unmet needs at home. If these SDoH are superimposed onto acute-on-chronic illness of hospitalized patients (1, 12–14), prolonged pediatric intensive care unit (PICU) care may be a result.

In the metropolitan Memphis, Tennessee and surrounding Shelby County areas, 25.3% of families live beneath the poverty line, which is twice the percentage for the state of Tennessee and 2.5 times that of the United States in general (15, 16). Due to the high percentage of poverty in these areas, we suspect that there are negative impacts on our patients from SDoH related to the economic instability and negative neighborhood attributes, which can impact healthcare delivery and overall patient outcomes. Evidence of neighborhood variation effects on negative health outcomes is consistent across the literature, despite heterogeneity of study designs, definitions, and locations (17). Health outcomes can occur on an income gradient and housing instability is associated with postponed medical care and missed medications (18, 19). Further, housing instability, food insecurity, and home health access are associated with delays in hospital discharge amongst CMC (19, 20). Infant bacterial infections and sepsis-related mortality are associated with health disparities and decreased socioeconomic and neighborhood quality (21–27).

We suspect that of all SDoH, high economic burden and neighborhood environment characteristics such as decreased housing quality, access to healthcare, food, and transportation could be key drivers of outcomes in our CMC patient population (28). As prolonged PICU length of stay (LOS) is associated with severity of illness in CMC, our study aimed to evaluate the effect of socioeconomically and neighborhood-driven factors on PICU LOS in CMC within the underserved community of Shelby County, Tennessee admitted to the pediatric critical care complex with severe sepsis. The secondary aim was to describe the CMC population admitted with severe sepsis. The central hypothesis of this study was that census tract-level socioeconomic and neighborhood SDoH are associated with longer PICU length of stay for CMC admitted with severe sepsis.

## Methods

### Study Design and Ethics

This retrospective single-center observational study was reviewed and approved by the Institutional Review Board at the University of Tennessee Health Science Center in Memphis, Tennessee.

### Patient Selection

For this study, patients aged 12 months up to 18 years with clinically relevant severe sepsis (severe sepsis identified using quantifiable data based on best evidence) were identified using a severe sepsis algorithm (29) incorporated into an existing electronic medical record (EMR), Cerner® Powerchart (Cerner Corporation, North Kansas City, Missouri). This alert mechanism continuously screens EMR-based physiologic data and laboratory results, and when previously defined criteria are met for SIRS and acute organ dysfunction (30), an electronic alert was generated and sent to a critical care smartphone with information regarding the alert characteristics. The patient's bedside nurse was also alerted, prompting an assessment by the primary team and a member of the PICU staff followed by recommendations for ongoing care or new therapies. The algorithm used in our hospital has been validated with a sensitivity of 90% and specificity of 96% (31, 32).

All hospital-wide severe sepsis alerts triggered between January 2019 through June 2020 were reviewed by a critical care clinician (HH) and two critical care nursing data analysts. Patients were determined to have clinically relevant severe sepsis based on clinical criteria (30, 33) and documented infection at the time of or within 24 h of the timestamped alert. Those meeting criteria for severe sepsis were further screened for PICU admission associated with the positive severe sepsis alert. The records of patients with PICU admissions were then examined for inclusion of patients with medical complexity and exclusion of those not defined as CMC and/or not admitted to PICU.

A study-specific definition for CMC was created and included patients with at least 2 of the following: a documented chronic disease or syndrome associated with functional limitations (for example cerebral palsy, chromosomal abnormalities, bronchopulmonary dysplasia/chronic lung disease, complex congenital heart disease), reliance on medical equipment or technology (such as gastrostomy tube, tracheostomy, home oxygen or ventilator, chronic central venous access for TPN or infusions), and/or high healthcare utilization. High utilization was defined as multiple visits to 2 or more pediatric subspecialty clinics in the 6 months prior to admission.

From this cohort, patients residing in Shelby County, Tennessee were selected for analysis based on available census tract data. Those with incomplete records were excluded from the analysis. Patients younger than 12 months were also excluded from the cohort as the electronic severe sepsis screening algorithm has not been validated for this age group. In the event of multiple admissions involving the same patient, each admission represented by a unique financial identification number (FIN) was treated as a separate encounter.

### Data Sources

We collected patient-level demographic data including medical history/diagnoses, age at PICU admission, sex, race, primary address, and insurance status directly from patient records. Our severe sepsis algorithm bins patients into the following age risk categories at the time of alert- 1–5, 6–12, and 13–18 years of age (29, 33). Race categories were White, Black/African American, and Other. The “Other” category included Latin/Hispanic, Asian, and Native American. Insurance categories were public and private. Public insurance is defined here as Medicaid or similar programs. Private insurance is defined here as commercial or military insurance. We also collected information regarding LOS, the source of sepsis, and the organ dysfunction associated with the episode of severe sepsis.

Next, we mapped the addresses of the 73 unique patients into 54 unique census tracts within Shelby County, Tennessee. Data for social vulnerability, environmental health hazards, and lead exposure risk for Memphis and Shelby County, Tennessee was obtained and visualized using PolicyMap®(34–36). Overall social vulnerability index (SVI), designed for disaster and disease outbreaks and indicates the negative impact of external factors on community health typically under hazardous conditions, was used as a major indicator of economic stability and neighborhood and built environment (37, 38). Each census tract is assigned an overall vulnerability level based on four categories: socioeconomic status, household composition, race/ethnicity/language, and housing and transportation. The SVI scale ranges from 0–1, with 1 representing the highest neighborhood-level social vulnerability. SVI was pre-classified into quartiles by the CDC as extremely low, low, moderate, and high categories.

Data pertaining to several variables reflecting neighborhood-level exposures were also analyzed. The Environmental Health Hazard Index (EHHI) from the US Department of Housing and Urban Development summarizes the potential for exposure to harmful toxins at a neighborhood level (35, 39). EHHI values range from 1 to 100 representing a percentile rank nationally, and higher values are representative of lower exposure risk and therefore better environmental quality of the neighborhood. As a surrogate of neighborhood quality, which includes increased pollutant exposure, neighborhood-level risk of lead exposure was evaluated using data from the Washington State Department of Health (40, 41). Lead exposure risk has been calculated based on the average age of buildings in a neighborhood and income-to-poverty ratio and we categorized neighborhoods as having low (bottom third), moderate (middle third), or high (top third) risk of lead exposure with 1 representing the lowest lead exposure and 10 the highest.

SDoH data corresponding to socioeconomic status (estimated median family income and percent of population below the poverty line) and household composition (percent of households headed by a single female with children) were obtained from the most recent US Census American Community Survey, 5-year estimate (42). Food access variables (distance to a farmers' market and percentage of housing units without a vehicle and located beyond 0.5 miles from a supermarket) were obtained from the US Department of Agriculture's food access research atlas (43). Neighborhood-level data pertaining to transportation (estimated percentage of housing units without a vehicle) and healthcare access (distance to the nearest hospital) were gathered from the University of Memphis Center for Applied Earth Science and Engineering Research (CAESER) (44).

### Outcome Measures

PICU LOS was identified as the primary outcome measure given that (1) high intensity therapies for this patient population occurs in the PICU and (2) our hospital discharges technology-dependent patients such as CMC directly from the PICU to home, capturing the duration of hospital stay.

### Statistical Analysis

Categorical data were summarized using frequency and percentages. The primary outcome PICU LOS was defined as the date from admission to date of discharge (or date of death). Patients who were not discharged, such as those who died in the PICU, were censored (45). To account for multiple encounters and time to event analysis, mixed effects Cox regression models were used to determine association or impact of each risk factor on PICU LOS for the bivariate analyses. For the multivariable mixed effects Cox regression model, a backward model selection approach was used to retain variables with a *p*-value < 0.05. A hazard ratio (HR) <1 indicates the risk of discharge is low or longer PICU LOS. All survival analyses were implemented using the statistical software R Studio. A *p*-value < 0.05 was considered statistically significant.

## Results

**Figure 1:**
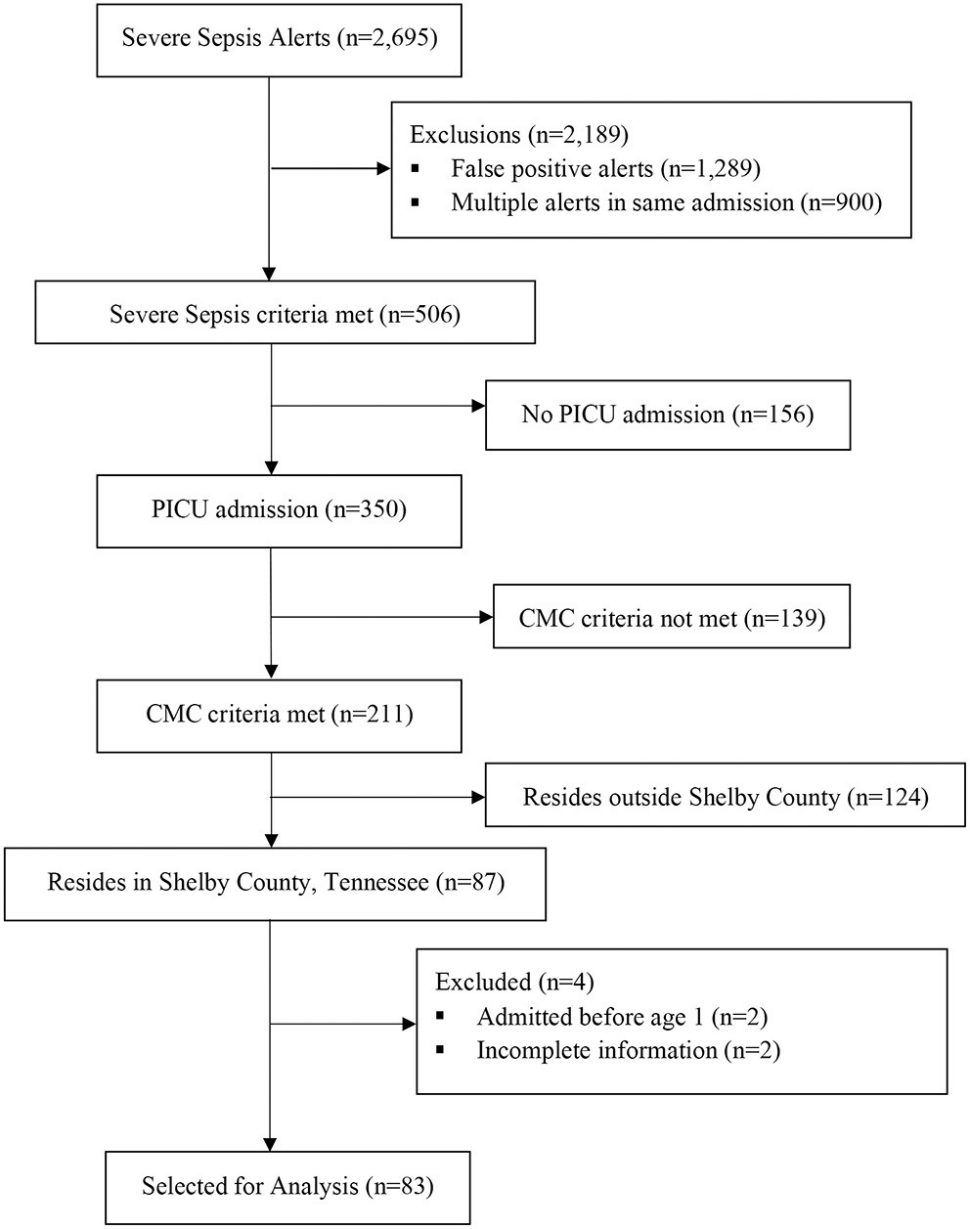
Patient encounter selection diagram.

Severe sepsis encounters are illustrated in Figure 1. Between January 1, 2019, and June 31, 2020, there were 2,695 severe sepsis alerts triggered in our hospital, 1,406 of which were determined to be true positives based on the predefined criteria. After reviewing each alert, 83 encounters representing 73 patients met inclusion criteria for the study.

### CMC Patient Demographics and Severe Sepsis Characteristics

Patient demographics can be viewed in Table 1. The median age of patients in the study was 4.1 years (IQR 1.96–12.02). There was a slight male predominance in the group (53 vs. 47%), and the racial diversity closely approximated that of Shelby County, Tennessee (68.7% Black, 18.1% White, and 13.3% Other). Most patients (85.5%) had public insurance. The median LOS in the PICU was 9.04 days (IQR 3.99–20.35) and mortality in the study population was 7.2%.

**Table 1:** CMC patient demographics.

**Age in years, *n* (%)**
1–5	46 (55.4)
6–12	15 (18.1)
13–18	22 (26.5)
**Race, *n* (%)**
Black/African American	57 (68.7)
White	15 (18.1)
Other	11 (13.3)
**Gender, *n* (%)**
Male	44 (53.0)
Female	39 (47.0)
**Insurance status, *n* (%)**
Public	71 (85.5)
Private	12 (14.5)

Table 2 describes the characteristics of medical complexity and severe sepsis at the time of severe sepsis alert. The majority (59%) of CMC patients in the study had more than one chronic condition or diagnosis. The most common conditions were neuromuscular (57.8%), followed by cardiovascular, respiratory, and genetic conditions (30.1%). Technology dependence was present in 88% of the CMC included in the study, with many (44.6%) relying on multiple pieces of equipment or technology. The most frequently observed modalities were gastrostomy tube (66.3%) and tracheostomy dependence (33.7%). Regarding healthcare utilization, 80.7% of patients included in the study received care from more than 2 subspecialists in the 6 months prior to admission.

**Table 2:** CMC co-morbidities and severe sepsis characteristics.

**CMC patient characteristics**	***n*** **(%)**
**Peri-alert source of infection**	
>1 source	9 (10.8)
Respiratory, viral	22 (26.5)
Respiratory, bacterial	32 (38.6)
Bloodstream	6 (7.2)
Urinary tract	9 (10.8)
Other^a^	11 (13.3)
Unknown^b^	12 (14.5)
**Pre-existing medical conditions**	
>1 condition	49 (59.0)
Neuromuscular	48 (57.8)
Cardiovascular	25 (30.1)
Respiratory	25 (30.1)
Gastrointestinal	7 (8.4)
Renal/genitourinary	10 (12.1)
Musculoskeletal	5 (6.0)
Oncologic	4 (4.8)
Genetic/chromosomal	25 (30.1)
Prematurity	26 (31.3)
Other (immune deficiency, transplant recipient, rheumatologic, endocrine condition)	17 (20.5)
**Pre-existing technology/equipment**	
>1 dependence	37 (44.6)
Gastrostomy	55 (66.3)
Tracheostomy	28 (33.7)
VP shunt	8 (9.6)
Central venous line	7 (8.4)
Home ventilator	10 (12.1)
Home oxygen	20 (24.1)
Other (baclofen pump, vagal nerve stimulator, dialysis catheter, pacemaker)	8 (9.6)
Patients with >2 subspecialists pre-PICU admission	67 (80.7)
**Present organ dysfunction at time of severe sepsis alert**	
>1 organ system	32 (38.6)
Respiratory	77 (92.8)
Cardiac	26 (31.3)
CNS	11 (13.3)
Renal	10 (12.1)
Hematologic	9 (10.8)
Hepatic	7 (8.4)
Mortality	6 (7.2)

a *Other source of infection—sources of infection not listed*.

b *Unknown source of infection—based on clinical and laboratory data outside of infection source microbiology*.

Respiratory infection (65.1%) was the most common source of sepsis, and 10.8% of patients had more than one source identified. Isolated respiratory failure was experienced by 56.6% of the study group, with an additional 36.1% of patients suffering respiratory failure along with dysfunction of another organ system (data not shown). Of the 83 encounters in the study, 3 required extracorporeal membrane oxygenation (ECMO) support, and 3 patients underwent new tracheostomy placement during the admission. As previously reported, 6 patients died.

### Association of Social Determinants of Health With LOS Among Pediatric CMC Patients With Severe Sepsis

SDoH of CMC patients with severe sepsis are described in Supplementary Table S1. The majority of patients are exposed to high SVI and lead exposure with access to healthcare, food sources, and transportation. Households consisted of 20% with a single female parent and 2% of households were non-English speaking. In a mixed effects Cox model, (Table 3), associations between PICU LOS vs. demographic and SDoH variables were analyzed. High and moderate SVI groups were associated with longer PICU LOS compared to extremely low SVI although not significant (Table 3). Neighborhood quality was comprised of lead exposure risk and environmental health index. Patient neighborhood quality in Shelby County was classified based on a score of 38 (range 4–71) and was not significantly correlated with PICU LOS. The length of PICU stay increases as the level of lead exposure risk increases but did not reach statistical significance (Table 3). The length of PICU stay decreases as the environmental health index increases, but this association is not statistically significant.

**Table 3:** Results of mixed effect survival models.

	***n***	**HR (95% CI)**	***p*** **-value**
**Demographics**			
**Age, years**			
1–5 (reference)	46	1	-
6–12	15	0.57 (0.25–1.28)	0.17
13–18	22	1.91 (0.98–3.72)	0.06
**Sex**			
Male	44	0.8 (0.5–1.26)	0.33
Female (reference)	39	1	-
**Race**			
Black (reference)	57	1	-
Other	26	0.93 (0.57–1.52)	0.77
**Insurance**			
Public (reference)	70	1	-
Other	13	1.09 (0.57–2.08)	0.79
**Overall social vulnerability index (SVI)^a^**			
Extremely low (reference)	18	1	-
Low	6	1.89 (0.74–4.87)	0.18
Moderate	18	0.98 (0.49–1.97)	0.95
High	41	0.90 (0.51–1.60)	0.72
**Risk of exposure to lead^b^**
1–4	19	1	-
5–7	30	0.89 (0.46–1.72)	0.72
8–10	34	0.72 (0.38–1.39)	0.33
**Environmental health index (1–100)^c^**	83	1.01 (1–1.03)	0.09
**Socioeconomic status**			
Estimated median income of a family	83	1 (1–1)	0.94
(USD)^d^			
% population below poverty rate	83	0.99 (0.98–1.01)	0.16

a *High (0.75–1.00), Moderate (0.50–0.75), Low (0.25–0.50), Extremely Low (0.00–0.25); Source: CDC (34, 37)*.

b *Low risk of lead exposure, 1–4; Moderate risk of lead exposure, 5–7; High risk of lead exposure; 8–10. Source: Washington State Department of Health (36, 40)*.

c *1–100 (Low–high index of environmental health hazard; 1–high health hazard, 100–low health hazard); Source, U.S. Dept. Housing and Urban Development (HUD) (35, 39)*.

d *Source, US Census ACS (2015–2019) (42)*.

Furthermore, there were no significant associations between other measures of SDoH and PICU LOS including estimated median income of a family, % population below poverty rate, estimated % housing units without vehicles, distance to the nearest hospital in miles, distance to the nearest farmer's market, estimated % of housing units without a vehicle and beyond 0.5 miles from a supermarket or grocery store, estimated % of all families that are headed by a single female with children, and estimated % of all people age 5 and older who were non-English speakers (data not shown). After following a backward model selection procedure and using *p* > 0.05 to remove non-significant variables, none of the variables remained in the mixed effects Cox multivariable model.

## Discussion

This is the first study to evaluate the association of socioeconomic and neighborhood-related SDoH with PICU LOS in the CMC population admitted with severe sepsis. While our results supported that SDoH among CMC presenting with severe sepsis were not associated with increased PICU LOS, we demonstrate several other key points. The study population was representative of the racial diversity in Shelby County, Tennessee (15). Patient age conformed to the bimodal peak of distribution observed in pediatric severe sepsis patients (46). Lastly, the median PICU LOS for CMC patients presenting with severe sepsis was 9 days, which is higher than the average LOS compared to other studies examining trends of all PICU children admitted with severe sepsis (7). Although Black race and public insurance are associated with PICU readmission of CMC following tracheostomy and gastrostomy tube placement (47), there was no significant difference attributable to gender, racial background, or insurance status in our study.

As childhood poverty is associated with increased PICU admission (48), SVI and specific neighborhood quality indicators (lead exposure risk and environmental health index) were the primary SDoH-related variables analyzed for association with PICU LOS. Although there were no significant associations found between socioeconomic and neighborhood factors in this study, there are trends worthy of discussion. Patients with high SVI and lower neighborhood quality were more susceptible to a longer PICU LOS than those in the other groups. This finding was not statistically significant, but larger studies with higher power might be warranted to evaluate SDoH effects on this outcome further. Other SDoH-related variables including socioeconomic status, access to transportation, healthcare, food sources, household composition, and language demonstrated no statistically significant associations with PICU LOS in our study. No significant associations between the SDoH variables and PICU LOS were found in our study population. We suspect the level of care provided at our facility mediates the impact of SDoH. Characteristics of care which could impede studying the impact of SDoH at our institution are the widespread education of severe sepsis across our institution, presence of a severe sepsis electronic alert mechanism, and rapid transfer of critically ill patients to the ICU.

There are other key factors that could not be evaluated in this study which may add a layer of complexity to SDoH of our CMC population and affect their outcomes such as PICU LOS. First, we did not consider the number of hospital readmissions as a potential outcome, which may be related to underlying abnormal or unstable functional status prior to previous PICU discharge (47, 49). Instability from a prior discharge with compounding SDoH could lead to an admission for critical illness including severe sepsis. Second, the availability of home health access or other healthcare utilization required by CMC was not considered as a risk factor. CMC often require extensive home health services involving additional economic resources which can compound pre-existing SDoH in any given home. Third, retrospective census-level data could mask individual poverty information of families with CMC and underestimate results, given that underserved children are at risk of higher PICU utilization (50). Lastly, other potential outcome measures such as mortality using the Pediatric Index of Mortality-2 (PIM-2) known to be associated with SDoH (51), severity of illness scoring measures for which we do not have sufficient data including the Pediatric Risk of Mortality Score III (PRISM III), were not evaluated in this study as some patients were not admitted to the PICU from the Emergency Department. Pediatric Logistic Organ Dysfunction-2 (PELOD-2) scores are not calculated at our hospital. Analysis of such outcome measures in association with SDoH may provide a more robust framework to further provide resources in the care of this patient population.

This study had several limitations. Sample size was limited by the incidence of severe sepsis and our strict inclusion criteria. Second, our hospital's location created some challenges for our team. Patients are referred to our center via hospitals around the Memphis metropolitan area, which includes 3 states and 9 counties, and beyond. CMC in the Memphis metropolitan area and Shelby County that died due to pediatric severe sepsis could not be accounted for in this study. Third, as community-level data is reported differently from county to county and state to state, we were limited in our ability to include more patients and to establish controls for comparison. Fourth, SDoH data was collected at a neighborhood-level, possibly limiting the ability to infer associations with individual PICU LOS. Individual screening of SDoH would provide information to better assess associations with individual PICU outcomes, including LOS. Lastly, we encountered documentation inconsistencies during our chart review process that prohibited analysis which controlled or corrected for variables such as illness severity and comorbidities that we feel would have strengthened our study.

## Conclusion

Incorporating SDoH data into clinical and public health decision-making processes enables precision prevention, treatment, and equity (28) and improves patient safety and quality of care. This study explored associations between social vulnerability and neighborhood-level SDoH in the health outcomes of CMC admitted to the PICU with severe sepsis. Although there are no associations between SDoH and CMC with severe sepsis in our exploratory analysis, we anticipate the results of this research will be used as a platform to conduct further confirmatory studies to advocate for increased resources for care of this population and generate new hypotheses using additional data collected from multiple settings at a higher geographical resolution for in-depth investigation of associations of PICU outcomes with SDoH.

## Data Availability Statement

The data analyzed in this study is subject to the following licenses/restrictions: The dataset from the EMR contains patient identifiers and is not publicly available. However, we did use public databases to search information, cited in the article. Requests to access these datasets should be directed to sshah7@uthsc.edu.

## Ethics Statement

The studies involving human participants were reviewed and approved by University of Tennessee Health Science Center. Written informed consent from the participants' legal guardian/next of kin was not required to participate in this study in accordance with the national legislation and the institutional requirements.

## Author Contributions

HH, AW, and SS conceptualized the study. AS-N, NA, and LC assisted with study design. HH reviewed chart and collected data. NA, TJ, FG, and LC completed the data analysis. HH and AW wrote the manuscript with assistance from NA and TJ and it was reviewed and edited by SS and AS-N. All authors reviewed the manuscript and approved it prior to submission.

## Conflict of Interest

The authors declare that the research was conducted in the absence of any commercial or financial relationships that could be construed as a potential conflict of interest.

## Publisher's Note

All claims expressed in this article are solely those of the authors and do not necessarily represent those of their affiliated organizations, or those of the publisher, the editors and the reviewers. Any product that may be evaluated in this article, or claim that may be made by its manufacturer, is not guaranteed or endorsed by the publisher.

## Supplementary Material

Supplementary Table S1 Social Determinants of Health among CMC with Severe Sepsis in Shelby County, Tennessee.
