# Supplementary material for: Analyzing Relationships Between Economic and Neighborhood-Related Social Determinants of Health and Intensive Care Unit Length of Stay for Critically Ill Children With Medical Complexity Presenting With Severe Sepsis
Source: Front Public Health. 2022 Apr 29;10:789999. doi: 10.3389/fpubh.2022.789999 (PMC9099028; doi:10.3389/fpubh.2022.789999)
Supplement: Supplementary Table S1 — Social Determinants of Health among CMC with Severe Sepsis in Shelby County, Tennessee. [file Table_1.pdf]

Table S1. Social Determinants of Health among CMC with Severe Sepsis in Shelby County, Tennessee

Social Determinants of Health	Evaluated Encounters (n)	Median (IQR)
**Overall Social Vulnerability Index (SVI)[a]**	83	
High	41	10.4 (3.32-22.13)
Moderate to High	18	7.77(3.24-17.68)
Low to Moderate	6	4.63 (4.18-7.14)
Low	18	8.52 (5.39-19.2)
Estimated median income of a family (USD)[b]	83	45504 (35752-78009)
% population below poverty rate[b]	83	26.5 (10.2-37.9)
**Neighborhood Quality**		
Risk of exposure to lead (1-10)[c]	83	
1	12	11.96 (5.08-25.46)
2	5	4.3 (0.96-4.95)
3	2	9.3 (0.92-17.68)
5	5	11.45 (7.14-12.51)
6	11	10.81 (4.45-23.16)
7	14	6.59 (2.61-9.95)
8	15	16.35 (3.24-38.4)
9	2	5.63 (3.42-7.83)
10	17	10.4 (5.97-20.35)
Environmental health hazard index (1-100)[d]	83	38 (29-49)
**Access to Transportation[e]**		
Estimated % of housing units without vehicles	83	6.81 (2.96-12.78)
**Access to Healthcare[e]**		
Distance to nearest Hospital (miles)	80	2.63 (1.87-3.63)
**Access to Food Sources[f]**		
Estimated % of housing units without a vehicle and beyond 0.5 miles from a supermarket or grocery store	83	0.06 (0.016-0.114)
Distance to nearest Farmers Market (miles)	80	3.03 (1.82-4.28)
**Parent Household Composition[b]**		

Estimated % of all families that are headed by a single female with children	83	20.11 (11.38-35.32)
**Minorities/Language**		
Estimated % of all people age 5 and older who were non-English speakers	83	2.27 (0.7-5.4)

[a]Source: CDC (33, 36)
[b]Source: US Census ACS (2015-2019) (39)
[c]1: low lead exposure, 10: high lead exposure; Source: Dept. Housing and Urban Development (HUD) (35,38)
[d]1 – high health hazard, 100 – low health hazard; Source: U.S. Dept. Housing and Urban Development (HUD) (as of 2015) (34,37)
[e]Source: CAESER (41)
[f]Source: USDA (2017) (40)
